# Eat Now or Later: Self-Control as an Overlapping Cognitive Mechanism of Depression and Obesity

**DOI:** 10.1371/journal.pone.0123136

**Published:** 2015-03-26

**Authors:** Gregory J. Privitera, Hannah K. McGrath, Brittany A. Windus, P. Murali Doraiswamy

**Affiliations:** 1 Department of Psychology, St. Bonaventure University, St. Bonaventure, United States of America; 2 Department of Psychiatry and Behavioral Sciences, Duke Institute for Brain Sciences and the Duke Brain and Society program, Duke University, Durham, United States of America; University of California, San Francisco, UNITED STATES

## Abstract

While overlapping neurobiological mechanisms are known, relatively little is known about how “self-control” and cognitive affective processing of rewards may also influence the bi-directional risk between obesity and depression. The objective of this study was to identify the extent to which “self-control,” measured using a delay discounting task is co-related to BMI and Depression diagnostic thresholds. A within-subjects counterbalanced design was used in which 92 participants (Mean±SD: BMI = 27.9±3.5, HAMD = 14.7±7.7) completed a series of clinical diagnostic, survey, and demographic questionnaires in a behavioral health laboratory setting. For the delay discounting task, participants chose between one large delayed reward and one successively smaller immediate reward for four food types (dessert, fried food, fruit, and vegetable). Results showed that delay discounting scores were predictive of BMI and depression with lower delay discounting scores associated with higher BMI and HAMD for the dessert (HAMD scores (β = -.197, p = .013), BMI (β = -.239, *p* < .001)) and fried food (HAMD scores (β = -.328, *p* = .001), BMI (β = -.166, *p* = .027)). Clinical significance was further evident when HAMD and BMI scores were converted to diagnostic thresholds. Only depression and/or atypical depressive symptoms were related to delay discounting scores with the fruit and vegetable. Thus, reduced cognitive affective self-control for impulsive food choices—particularly for “comfort foods” high in fat and sugar—appears to be a shared cognitive mechanism for both conditions perhaps contributing to the high prevalence of co-morbid mood disorders and weight gain.

## Introduction

Delay discounting and delayed gratification represent two indices of impulsive behavior; each measure quantifies the “impulsive” tendency of a participant to discount the value of distant rewards in favor of immediate rewards. The Cognitive Affective Processing System (CAPS) theory of self-regulation, supported by functional magnetic resonance imaging (fMRI) evidence [1], posits that the greater that participants delay their gratification, the more they may use “cold cognition” decision making strategies (prefrontal cortext, top down processing) versus “hot cognition” strategies (bottom up processing, emotional), which would be associated with greater delay discounting. Concomitantly, the severity of delay discounting has been linked to dopamine receptor polymorphisms [2], BMI scores [3] and depression [4].

Among adults, there has been a marked rise is the comorbidities and prevalence of depression [5,6] and obesity [7, 8], making it important to understand how these two disorders may be related. Although some studies show a negative or no association in the prevalence of depression and obesity [9–11], most evidence suggests at least a positive correlational [12–14] and even a possible causal relationship [15] between the observed rise in both, with the largest association between depression and obesity observed for those with atypical depression, which is depression that includes symptoms of increased appetite and weight gain [16]. While many studies have investigated the extent to which these two disorders are related, relatively fewer have specifically tested possible overlapping mechanisms for the apparent bi-directional risk between obesity and depression.

Neurobiological evidence for an overlap between depression and obesity points to brain-reward regions, such as dopaminergic pathways [17–20], which can enhance positive mood, yet also lead to increased intake of “comfort” foods, which are foods that tend to be high in sugar and fat [21,22]. Recent evidence indicates that reporting elevated depression symptoms is associated with greater emotional eating, specifically among obese adults with elevated depression symptoms [23]. Consuming [21], viewing [17–24], and even expressing “comfort” foods in art [25], can enhance mood and promote increased activity in dopaminergic pathways, which can lead to obesity.

One possible mechanism that may underlie these findings is that increased activity in dopaminergic pathways is related to increased impulsivity and a corresponding reduced ability to control impulses for many behaviors, including smoking [26,27], drug abuse [28], feeding [29], and depression-induced attempted suicide [30]. A common measure of impulsivity in humans is delay discounting in which participants are given the choice between one large delayed reward and one successively smaller immediate reward [31]. To quantify impulsivity indifference points are typically computed [32], which is the point at which participants switch from choosing a smaller immediate reward to choosing a larger delayed reward; choosing the smaller immediate reward is the impulsive choice; choosing the larger delayed reward is the self-controlled choice [33]. In the present study, a single indifference point was computed for each participant, to limit the number of trials each participant completed, and so that a single dependent measure could be compared across categorical groups.

While much is known about the overlapping neurobiological mechanisms between depression and obesity, relatively little is known about the influence of “self-control” on the bi-directional risk between obesity and depression. In the present study, we specifically tested “self-control” using food items as choices in the delay discounting procedure to evaluate its relationship with depression and BMI characteristics. One hypothesis is that those with depression have a reduced ability to control their food choice specifically for “comfort” foods that can stimulate these brain reward regions. Thus, those with depression may make more impulsive food choices in a delay discounting task. A corollary to this hypothesis is such a finding may be most prevalent among those with atypical depression [34] and participants who are obese, which was tested here with an analysis for the clinical and statistical significance of the relationship between self-control of food choice (measured by delay discounting), obesity (measured by BMI), and depression (measured by the HAMD [35]) for each of four food types (a dessert, fried food, fruit, and vegetable).

## Method

### Participants

All participants were adults who signed a written informed consent. The St. Bonaventure University Institutional Review Board (IRB) approved the protocol for this study. IRB Approval number: IRBSP14-08. A total of 92 university undergraduate students (32 men, 60 women) were recruited through university classroom visits and sign-up sheets. The following participant variables are summarized by sex and depression category in Table 1: height, weight, age, and BMI. In an initial screening phase, participants reported that they were in general good health with no significant physical or doctor diagnosed food allergies, pregnancy, or dietary restrictions. Subjects with and without current depressive symptoms were permitted to enter the study. None of recruited participants were on psychiatric medications. Participants were told during the initial screening phase not to eat within two hours of the study. Because hunger states can influence food choice and intake [36, 37], participants who ate within two hours of the study were excluded from data analyses; hunger was further added as a covariate in statistical analyses. All participants identified that they were familiar with, had consumed, and generally liked the foods for which they were asked to evaluate in the delay discounting task.

**Table 1:** Participant characteristics by sex and depression diagnosis criteria.

			Height (cm)		Weight (kg)		Age (years)		BMI (kg/m^2^)	
HAMD-17	Sex	*N*	*M*	SD	*M*	SD	*M*	SD	*M*	SD
**Normal (0–7)**	**M**	8	175.2	10.9	82.2	12.1	19.5	1.3	26.8	3.0
	**F**	12	169.7	10.8	78.0	16.5	19.4	1.2	26.9	3.9
**Mild-Moderate (8–18)**	**M**	14	177.4	8.4	86.5	9.1	19.7	1.3	27.6	3.1
	**F**	27	164.9	6.1	76.5	12.8	19.2	1.0	28.0	3.8
**Severe-Very Severe (> 18)**	**M**	10	179.3	6.8	88.9	11.3	19.5	1.2	27.6	3.1
	**F**	21	160.5	6.5	74.4	9.4	19.8	1.1	28.9	3.1

Values given by sample size (N), means (M), and standard deviations (SD).

### Procedures

**Fig 1:**
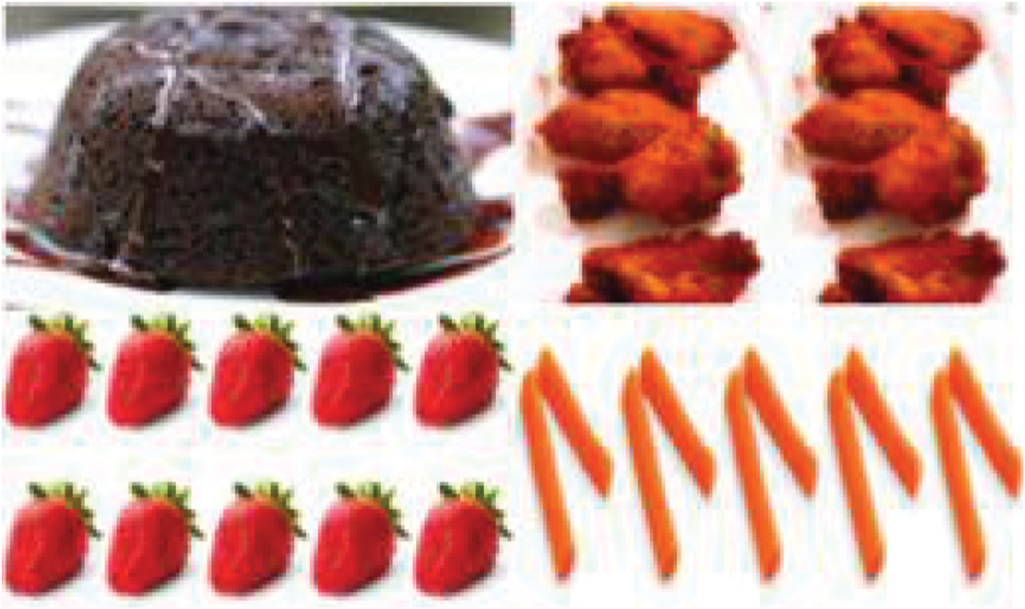
Food images shown for each delay discounting task. Each image shows the full portion. Next to each image was also a comparison photo of a single portion with either 1/10 of a slice of the cake, one chicken wing, one strawberry, or one carrot stick, respectively.

Participants were seated one at a time at tables in a quiet room. Participants first completed the delay discounting task, which was adapted for use with food items. The delay discounting task asked participants to choose varying portions of a piece of cake (dessert), chicken wings (fried food), strawberries (fruit), and carrot sticks (vegetable). Thus, participants completed the delay discounting task four times (one time for each food). Fig. 1 shows the food image for each delay discounting task. For each food type, participants were told, “In the following task, assume you could have each of the foods you will see pictured.” They were then given the option to choose to wait four hours for a whole portion (ten servings) or choose incrementally smaller portions, then incrementally larger portions. The *indifference point* was measured as the average of the points at which a participant switched from choosing a larger portion of food later to a smaller portion of that food now [31]. To clarify each choice, an image of each serving option accompanied each choice. To clarify further the procedure, a participant began with being offered a whole portion now or a whole portion in four hours, then nine servings now or the whole portion in four hours, and so on until offered one serving now. At one serving, the immediate option increased again until the participant was again offered the whole portion now or the whole portion in 4 hours. Because hunger states would change during the course of the task if the foods were consumed, participants were only allowed to choose foods, not eat them. Also, food images, and not actual foods were used to avoid confounds regarding the influence/changes in the sensory properties of the foods during the course of the task [38]. Lower indifference points indicated less self-control/more impulsive food choices [33].

Once the delay discounting task was completed, demographic and depression data were recorded. To measure depression, participants were evaluated using the Hamilton Depression Rating Scale (HAMD) [35] in which the first 17 (of 21) items are scored. For categorical analyses, 0–7 was normal, 8–18 was mild-moderate depression, and greater than 18 was severe depression. To determine atypical symptoms of depression, the four additional items from the HAMD were summed with higher scores indicating greater atypical symptoms. BMI was computed to determine obesity diagnosis. Measures of depression and BMI followed the delay discounting task to reduce the likelihood that such measures would influence responses on the delay discounting task. Once finished, participants were given a debriefing form and dismissed. Those with elevated depression scores were referred for mental health follow up, as appropriate.

### Statistical Analysis

To evaluate the clinical significance of depression and BMI on delay discounting scores, a 3 × 3 × 2 multivariate analysis of covariance (ANCOVA) was computed with depression categories (normal, mild/moderate, severe/very severe) BMI categories (lean: < 25.0, overweight: 25.0 to 30.0, obese: > 30.0), and sex (male, female) as the between-subjects factors, and delay discounting scores for the dessert, fried food, fruit, and vegetable as the dependent variables. Self-reported ratings of hunger were included as a covariate. While the images of the foods likely varied in color, calorie count, dietary history, economic cost, and other ways, delay discounting scores were separately analyzed for each food type. Thus, comparisons were made within- (not between-) food categories. For this reason, it was not necessary to hold these factors constant across (i.e., between) food types. The experimentwise alpha was corrected for all post hoc analyses using a Bonferroni procedure, and the 95% CI is reported for pairwise comparisons. For each confidence interval, the null hypothesis tested was 0 difference between two groups. An effect was evident when the confidence interval did not envelop 0.

To further evaluate the significance of depression and BMI on delay discounting scores, a multiple regression analysis was computed with BMI and HAMD scores assessed. A total score for atypical items on the HAMD was also calculated for each participant, and a multiple regression analysis was computed for each food type, with BMI, HAMD scores, and the total score for items used to assess atypical depression included as predictor variables; delay discounting scores were the dependent variables. Raw scores, and not categories, were assessed for BMI and HAMD in the regression analysis. All tests were computed at a. 05 level of significance.

## Results

Of the 92 participants, mean/SD age was 19.5±1.2 years, mean height was 167.6±9.9 cm, and mean weight was 78.5±12.4 kg. BMI scores ranged from 20.1 to 34.1 kg/m^2^ with a mean/SD of 27.9±3.5 kg/m^2^. HAMD-17 scores ranged from 4 to 32 with a mean/SD score of 14.7±7.7. Table 2 depicts delay gratification scores by depression category. For all analyses reported here, there were no significant effects of sex (*p* >. 07 for all tests); the covariate for the ANCOVA was evaluated at the following value: hunger rating = 3.12.

**Table 2:** Delay discounting scores for participants in each category of depression.

		Delay Discounting Scores							
		Dessert		Fried Food		Fruit		Vegetable	
HAMD-17	*N*	*M*	SD	*M*	SD	*M*	SD	*M*	SD
**TOTALS**	92	4.82	2.96	4.32	2.65	4.34	2.75	3.76	2.81
**Normal (0–7)**	20	7.03	1.99	6.95	1.76	5.65	2.13	4.85	2.66
**Mild-Moderate (8–18)**	41	4.99	2.76	4.18	2.14	4.54	2.88	3.83	2.81
**Severe-Very Severe (> 18)**	31	2.34	2.20	2.60	2.24	2.65	2.65	3.16	2.97

The analyses of delay discounting scores for depression and BMI were evaluated using both continuous and categorical cut-points on HAMD. Values given by sample size (N), means (M), and standard deviations (SD). Details of analyses are given in text.

### Dependent variable: Dessert

**Fig 2:**
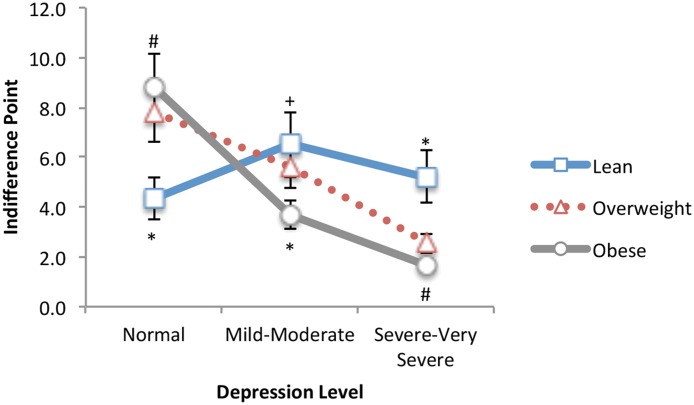
Dessert Food: Delay discounting as a function of BMI and depression level for a dessert food. At severe to very severe depression levels, participants who are overweight/obese show significantly greater impulsivity/lower control for a choice of a dessert food compared to lean weight participants. *Significantly different from all other groups (*p* <. 01). #Significantly different from the Lean group only (*p* <. 05). +Significantly different from the obese group only (*p* <. 05).

With dessert as the food type, the ANCOVA showed a significant main effect of depression, F(2, 73) = 15.55, *p* <. 001 (*R* ^*2*^ = .30), and as shown in Fig. 2, a significant depression × BMI interaction, F(4, 73) = 7.76, p <. 001 (*R* ^*2*^ = .30). The main effect showed that delay discounting scores were significantly different for each pairwise comparison: normal-moderate (95% CI 0.42, 3.65), moderate-severe (95% CI 1.24, 4.06), and normal-severe (95% CI 2.99, 6.39). The main effect showed that more severe depression was associated with lower delay discounting scores, or more impulsive food choices. To assess the depression × BMI interaction, a one-way between-subjects ANCOVA was computed for BMI at each level of depression; hunger ratings were again included as a covariate. BMI was a significant factor for those with normal, F(2, 16) = 42.94, *p* <. 001 (*R* ^*2*^ = .84), moderate, F(2, 37) = 4.11, *p* = .025 (*R* ^*2*^ = .18), and severe depression, F(2, 27) = 9.02, *p* = .001 (*R* ^*2*^ = .40). Post hoc analyses showed that, compared to lean weight participants, obese participants with moderate (lean-obese: 95% CI 0.08, 5.72), and severe depression (lean-obese: 95% CI 1.75, 7.04) had significantly lower delay discounting scores (more impulsive choices). For the no depression (normal) group, the opposite effect was observed: delay discounting scores were lower for lean compared to obese participants (lean-obese: 95% CI -3.11, -5.75). A multiple regression analysis further showed a significantly predictive model, F(3, 88) = 61.58, *p* <. 001 (*R* ^*2*^ = .68), with HAMD scores (β = -.197, *p* = .013), BMI (β = -.239, *p* <. 001), and scores for atypical symptoms of depression (β = -.637, *p* <. 001) each independently predicting significant variance in delay discounting scores with dessert as the food type.

### Dependent variable: Fried foods

**Fig 3:**
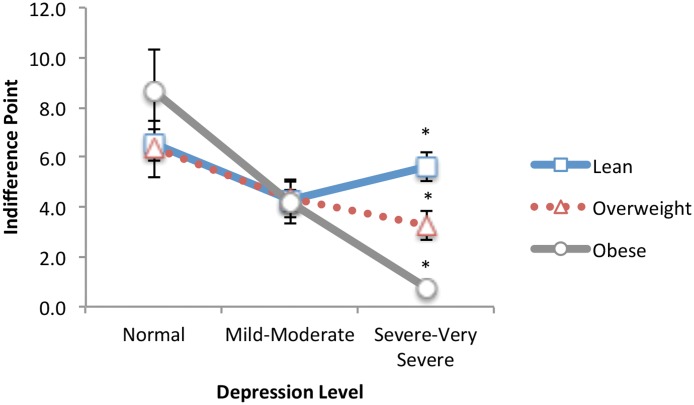
Fried Food: Delay discounting as a function of BMI and depression level for a fried food. At severe to very severe depression levels, participants who are overweight/obese show significantly greater impulsivity/lower control for a choice of a fried food compared to lean weight participants. *Significantly different from all other groups (*p* <. 01).

With fried food as the food type, the ANCOVA showed a significant main effect of depression, F(2, 73) = 22.73, *p* <. 001 (*R* ^*2*^ = .38), and as shown in Fig. 3, a significant depression × BMI interaction, F(4, 73) = 4.44, *p* = .003 (*R* ^*2*^ = .20). Same as for desserts, the main effect showed that delay discounting scores were significantly different for each group: normal-moderate (95% CI 1.37, 4.16), moderate-severe (95% CI 0.37, 2.81), and normal-severe (95% CI 2.88, 5.82). Using the same test to assess the depression × BMI interaction, BMI was again a significant factor for those with severe depression, F(2, 27) = 14.65, *p* <. 001 (*R* ^*2*^ = .52), but not for those with normal, *p* = .06, and moderate depression, *p* = .96. Post hoc analyses showed that for those with severe depression, obese participants had significantly lower delay discounting scores (more impulsive choices) than lean weight participants (lean-obese: 95% CI 2.17, 6.41). A multiple regression analysis further showed a significantly predictive model, F(3, 88) = 35.27, *p* <. 001 (*R* ^*2*^ = .55), with HAMD scores (β = -.328, *p* = .001), BMI (β = -.166, *p* = .027), and scores for atypical symptoms of depression (β = -.455, *p* <. 001) each independently predicting significant variance in delay discounting scores.

### Dependent variable: Fruit

With fruit as the food type, the ANCOVA showed only a significant main effect of depression, F(2, 73) = 5.27, *p* = .007 (*R* ^*2*^ = .13). The main effect showed that delay discounting scores were significantly lower for those with severe depression compared to both other groups: moderate-severe (95% CI 0.34, 3.18), and normal-severe (95% CI 0.94, 4.40). The multiple regression analysis further showed a significantly predictive model, F(3, 88) = 14.47, *p* <. 001 (*R* ^*2*^ = .33), with HAMD scores (β = -.281, *p* = .014), and scores for atypical symptoms of depression (β = -.364, *p* <. 001) independently predicting significant variance in delay discounting scores, but not BMI (β = .046, *p* = .609).

### Dependent variable: Vegetable

The ANCOVA showed no significant effects with the vegetable as the food type (*p* >. 06 for all tests), suggesting that “self-control” of food choice for a vegetable is not related to the clinical diagnosis of depression or BMI. However, the multiple regression analysis showed a significantly predictive model, F(3, 88) = 4.64, *p* = .005 (*R* ^*2*^ = .14), with scores for atypical symptoms of depression (β = -.274, *p* = .03) independently predicting significant variance in delay discounting scores, but not HAMD scores (β = -.097, *p* = .445), nor BMI (β = -.119, *p* = .245).

## Discussion

To our knowledge, this is the first prospective study to test the relationship between impulsivity in food choice and the presence of obesity and depression. The results show an interesting pattern that, when taken together, suggests that delay discounting, or the ability to control impulsive food choices, is related to the bi-directional risk of depression and obesity (BMI) specifically for “comfort foods,” such as high fat and high sugar foods (i.e., the dessert and fried food). Clinical significance was evident for BMI and depression with lower indifference points (i.e., more impulsive food choices) observed for moderately and severely depressed participants who were obese. Using fruits, clinical significance was evident for depression only, with lower indifference points evident among those who were severely depressed compared to those with scores in the normal range—this is consistent with recent studies showing enhance positive emotion after viewing fruits and expressing fruits in art [24,25]. No clinical significance was evident using the vegetable, as would be expected based on evidence showing a relationship between reward discounting and impulsive behavior for patients with a mood disorder [39]. Taken together, these findings show that delay discounting or “self-control” is related to the clinical severity of BMI and depression, with reduced self-control related to both disorders specifically for choices of comfort foods (i.e., the dessert and fried food).

As “comfort foods” become increasingly available in an environment, as it has over the last half century [40], one expectation is that those with depression will increasingly seek out those foods for comfort, or to improve mood [19,41]. Consumption of the comfort foods, often high in fat and sugar, can lead to improved emotional states [21], and yet intake of these foods in response to emotions has been linked to the rising rates in obesity [42,43]. As expected, those with the least self-control for choosing comfort foods would thus be at the greatest risk for both disorders. Consistent with this expectation, our findings show that those who are obese and severely depressed had the least self-control for choosing a dessert and fried food, as measured by a delay discounting task. Given that food choice and food intake are related [38], the findings presented here provide one possible explanation for the bi-directional relationship between obesity and depression—both are associated with reduced self-control for foods that simultaneously contribute most to obesity, and can have the effect of making a person feel better when depressed [18,22–25].

Interestingly, the analysis for statistical significance showed that for all food types, atypical symptoms of depression were predictive of delay discounting, with greater atypical symptoms being associated with less self-control or more impulsive food choices for the dessert, fried food, fruit, and vegetable. A national survey of 43,093 adults found that the prevalence of major depression with atypical features was about 40% higher than that of depression without atypical features [44]. In this study, variables that predicted atypical depression appear to mirror the growth of obesity and over eating in our society as a whole highlighting the potential role of brain-environment interactions. In addition, recent evidence suggests an association between preference for sweet taste and depression in obese patients [45], and other evidence suggests that the hedonic response to sweet taste is associated with elevated sensitivity to the mood altering effects of sweet-tasting foods [46]. The findings presented here suggest further that depression is associated with reduced self-control for these types of foods that taste sweet, such as a fruit.

Traditionally, choosing the smaller immediate reward is interpreted as the impulsive choice; choosing the larger delayed reward is the self-controlled choice [33]. Using food types as the target choice, it could be argued that choosing an immediate smaller food portion is instead the more self-controlled choice; choosing the delayed larger portion is the impulsive choice. This could explain the paradoxical finding that among those without depression, lean participants showed greater impulsivity for the dessert food than those who were overweight or obese. However, this paradoxical finding was the only anomaly in the data that would be consistent with such an explanation across all food types. For all other food types, self-control showed no significance across BMI categories that would be expected based on this alternate interpretation. Instead, with “comfort foods” as the target choice, the results for those with severe depression are most consistent with the standard interpretation that the immediate reward is the impulsive choice, and the larger delayed reward is the self-controlled choice. Still, while our findings overall are most consistent with the standard interpretation for this task [31, 33], the paradoxical finding in our study suggests that future studies can and should test the possibility of an alternate interpretation for a delay discounting task when foods are used as the target choice.

A key limitation for the present study is that a hyperbolic discounting curve [31] could not be analyzed because only one indifference point was computed for each food type. In the present study, the immediate reward was not varied at different delays, as is most typical [31]. Instead the delay was held constant and the magnitude of the immediate reward was varied. This procedure allowed for a single dependent variable, or indifference point, to be compared across groups and to minimize the number of times participants completed the same task. Future studies are needed to specifically compare the results reported here to studies in which multiple indifference points are plotted along a hyperbolic discounting curve.

Some additional limitations can be identified here. First, whether reduced self-control causes increased risk of depression and obesity, or vise versa cannot be determined, although the pattern of results shows that these factors are related. In addition, the delay discounting task included only four foods—one food for each category. One food was used in each category to reduce the time it would take to complete the delay discounting task and minimize the number of times participants were asked to complete the same task (to avoid a response set pitfall due to repeatedly completing the same task). Two key limitations follow. First, differences cannot be compared between the food types. The foods vary in many characteristics, therefore it is important that future work look at which specific characteristics of the food themselves (e.g., calories, portion size) may lead to effects of delay discounting. Also, it is not possible to generalize beyond the food types used in this study, although such a possibility can and should also be tested. Because the fried food was a meat food, other studies could look at vegetarian fried foods (such as French fries) to determine the generalizability across diet types. Other participant characteristics not tested in this study include ethnicity and participant motivation to manage body weight. Whether these additional factors may interact with analyses reported here cannot be evaluated, but can and should be tested in future studies.

Although the power for this study was satisfactory [47] (i.e., for tests of all dependent variables, the observed power was greater than. 80), it would be advantageous to conduct these types of studies with population-based samples to strengthen generalization across the full spectrum of BMI and depression diagnosis criteria. Likewise it would be important to examine delay discounting in other psychiatric conditions such as bipolar disorder and schizophrenia, which have also been linked to obesity as well as to examine the effects of psychotropic drugs such as antidepressants and antipsychotics. Given the plausible role of hot cognition circuits, it would also be of interest to test whether focal neuromodulation of specific circuits might help ameliorate maladaptive delay discounting.

Taken together, these findings are consistent with a growing body of research showing that the marked rise in the comorbidities and prevalence of obesity and depression are related or bi-directional [5–8]. These findings further extend this body of research by showing for the first time that alterations in cognitive affective processing (i.e., a reduced ability to control impulsive food choices) specifically for “comfort foods,” (i.e., a dessert and fried food) may be related to the bi-directional risk of depression and obesity. Future studies will test the limitations identified here and may open up new avenues for treating comorbid obesity and depression.
